# Inhibition of Polyphosphate Degradation in *Synechocystis* sp. PCC6803 through Inactivation of the *phoU* Gene

**DOI:** 10.4014/jmb.2311.11046

**Published:** 2024-01-08

**Authors:** Han-bin Ryu, Mi-Jin Kang, Kyung-Min Choi, Il-Kyu Yang, Seong-Joo Hong, Choul-Gyun Lee

**Affiliations:** 1 Department of Biological Engineering, Inha University, Incheon 22212, Republic of Korea; 2 Industry-Academia Interactive R&E Center for Bioprocess Innovation, Inha University, Incheon 22212, Republic of Korea

**Keywords:** Cyanobacteria, *Synechocystis*, bio-fertilizer, polyphosphate, *phoU*

## Abstract

Phosphorus is an essential but non-renewable nutrient resource critical for agriculture. Luxury phosphorus uptake allows microalgae to synthesize polyphosphate and accumulate phosphorus, but, depending on the strain of algae, polyphosphate may be degraded within 4 hours of accumulation. We studied the recovery of phosphorus from wastewater through luxury uptake by an engineered strain of *Synechocystis* sp. with inhibited polyphosphate degradation and the effect of this engineered *Synechocystis* biomass on lettuce growth. First, a strain (Δ*phoU*) lacking the *phoU* gene, which encodes a negative regulator of environmental phosphate concentrations, was generated to inhibit polyphosphate degradation in cells. Polyphosphate concentrations in the *phoU* knock-out strain were maintained for 24 h and then decreased slowly. In contrast, polyphosphate concentrations in the wild-type strain increased up to 4 h and then decreased rapidly. In addition, polyphosphate concentration in the *phoU* knockout strain cultured in semi-permeable membrane bioreactors with artificial wastewater medium was 2.5 times higher than that in the wild type and decreased to only 16% after 48 h. The biomass of lettuce treated with the *phoU* knockout strain (0.157 mg P/m^2^) was 38% higher than that of the lettuce treated with the control group. These results indicate that treating lettuce with this microalgal biomass can be beneficial to crop growth. These results suggest that the use of polyphosphate-accumulating microalgae as biofertilizers may alleviate the effects of a diminishing phosphorous supply. These findings can be used as a basis for additional genetic engineering to increase intracellular polyphosphate levels.

## Introduction

In modern agriculture, nitrogen and phosphorus fertilizers are essential for the growth, survival, and reproduction of crops [1]. Nitrogen, which is synthesized through the Harbor-Bosch process, has significantly contributed to increasing agricultural productivity and reducing hunger worldwide [2]. Unlike nitrogen fertilizers, which incorporate ammonia synthesized from nitrogen that is abundantly available in the atmosphere, phosphorus fertilizers are obtained from phosphorus rock [3], which is expected to be scarce worldwide within 30 to 300 years [4]. Phosphorus is a limiting factor for crop growth, and global crop yields are critically dependent on phosphorus supply. Microalgae have attracted attention as biofertilizers to overcome phosphorus shortage. Phosphorus is an essential nutrient for the survival of microalgae because it is a major component of nucleic acids, phospholipids, and ATP [5].

Microalgae possess metabolic mechanisms that allow them to survive in extreme environments. Luxury uptake is a mechanism through which excess phosphorus is absorbed for the organisms to adapt to phosphate-deficient conditions [6]. If microalgae under phosphate-deficient conditions are exposed to excess phosphate, they accumulate 0.41 to 3.16% or more of their dry weight [7]. A large amount of absorbed phosphorus accumulates in cells in the form of polyphosphate (poly-P) [8]; therefore, microalgal biomass could serve as a phosphate fertilizer. However, most microalgae degrade the accumulated poly-P after a certain period. The duration of accumulation differs for each species of microalga, ranging from 1 to 4 days [6]. Degradation of the accumulated poly-P is regulated by the PhoU negative regulator protein. The presence of high concentrations of phosphates in external environments is known to inhibit the expression of proteins that absorb phosphates into cells via the PhoU protein. The expression of polyphosphate exopolyphosphate (PPX) proteins induced by PhoU increases to degrade poly-P [9]. Accordingly, poly-P degradation can be prevented if PhoU is knocked out.

A study of *phoU* gene knock-out was conducted in *Escherichia coli* and *Synechocystis* sp. PCC6803 (Syn6803), and accumulation of poly-P were confirmed in both the strains [10]. Subsequently, a Syn6803 *phoU* knockout strain (Δ*phoU*) was used to remove phosphorus from wastewater [11]. Recently, studies have been conducted to produce the biodegradable polymer poly (hydroxybutyrate) using the Δ*phoU* strain [12]. Phosphorus in microalgae stored in the form of intracellular phosphate is biosoluble and can be used as a biofertilizer in agriculture [13]. In this study, we aimed to use poly P-accumulated Syn6803 as a fertilizer that inhibits the rapid degradation of poly-P through knockout of the *phoU* gene in Syn6803.

In this study, Syn6803 was used to generate an engineered strain with a reduced rate of poly-P degradation. Analyses focused on the accumulation and degradation of poly-P in the Δ*phoU* strain for use as a phosphorus fertilizer. Development of microalgal biofertilizers could reduce the dependence on synthetic fertilizers, in addition to reducing carbon dioxide emissions, and provide a sustainable supply of nitrogen and phosphorus for agricultural use.

## Materials and Methods

### Algal Culture Conditions and Biomass Determination

The Syn6803 strain was obtained from the Pasteur Culture Collection (France). For liquid culture, 30 μmol/m^2^/s fluorescent lamps (Model DULUX L, 36 W/865, OSRAM Korea) were irradiated continuously in a 250 ml Erlenmeyer flask containing 100 ml BG-11 medium. The BG-11 medium was prepared according to a previous protocol [14], with a slight modification (10 mM glucose was added). The culture was incubated at 30°C with constant shaking at 120 rpm; 150 μg/ml spectinomycin was added to the culture of mutant strains. For solid culture, 1.5% agar powder and 1 mM Na_2_S_2_O_3_·5H_2_O were added.

The cells were cultured for 72 h in BG-11 medium from which phosphorus was removed under the same conditions as described above to induce poly-P accumulation. Thereafter, 1.12 mM K_2_HPO_4_ was added to observe the accumulation and degradation of poly-P.

The environmental conditions for the artificial wastewater experiment were the same as described above, except that the cells were cultured in the modified N8 medium. The N8 medium was prepared by dissolving 9.89 mM KNO_3_, 5.44 mM KH_2_PO_4_, 1.48 mM Na_2_HPO_4_·2H_2_O, 0.20 mM MgSO_4_·7H_2_O, 0.09 mM CaCl_2_·2H_2_O, 0.027mM Fe-Na EDTA, and trace minerals in 1 L distilled water. Trace minerals were delivered in a stock solution containing 11.13 μM ZnSO_4_·7H_2_O, 30 μM MnCl_2_·4H_2_O, 7 μM CuSO_4_·5H_2_O, and 5.39 μM Al(SO_4_)_3_·8H_2_O.

Cell concentrations were measured using a Coulter Counter (Multisizer 4; Beckman Coulter Inc., USA). Fresh cell weight (FCW) was determined by calculating cell concentration, cell size distribution, and average cell size through various data compiled using the Multisizer 4 software and exported to an Excel spreadsheet [15].

### Transformant Construction

**Fig. 1:**
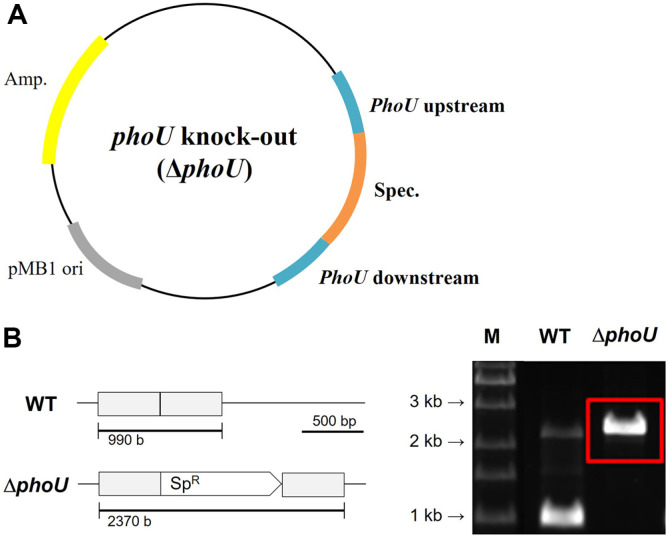
A. Plasmid constructs used to transform *Synechocystis*; B. Genetic map and PCR analysis of *Synechocystis* wild-type (WT) and Δ*phoU* transformants. Δ*phoU*: Syn6803 *phoU* knockout strain, Sp^R^: spectinomycin resistance cassette.

Syn6803 was transformed using homologous recombination. The locus name of the *phoU* gene is *slr0741*; the Syn6803 sequence was obtained from the NCBI database. Three DNA fragments- fragment 1 upstream of *slr0741*, spectinomycin antibiotic resistance gene *aadA*, and fragment 2 downstream of *slr0741*- were produced to create a *phoU* knockout cassette (Fig. 1A). An Infusion Cloning Kit (Takara, Japan) and PCR were used to ligate the three DNA fragments. Nucleic acid sequences of the primers used for infusion cloning are listed in Table 1. The spectinomycin resistance gene was used as the selection marker. Natural transformation was performed as described previously [16]. Transformants were picked by culturing the obtained colonies from one-eighth of the concentration of 50 μg/ml to complete concentration. *phoU* knockout mutants were cultured in BG-11 medium supplemented with spectinomycin to extract gDNA. Transformants were confirmed by gDNA extraction and PCR amplification. Band sizes of the wild type and the transformant were compared by 1D-electrophoresis (Fig. 1B).

### Measurement of Poly-P Accumulation and Phosphate in Media

The concentration of poly-P was measured using a spectrophotometer after staining poly-P in toluidine blue [13]. The microalgal culture (10 ml) was centrifuged at 2,350 ×*g* for 5 min to remove the supernatant, and the cell pellet was washed twice with 500 μl sterilized water. After measuring the weight of the cell, 600 μl sterilized water was added to resuspend the cells. After ultrasonication for 5 min, the mixture was placed in a water bath at 100°C and boiled for 2 h. Thereafter, it was cooled at 25°C, and 600 μl of a 24:1 (v/v) chloroform:isoamyl alcohol mixture was added. After centrifugation at room temperature for 15 min, the supernatant was transferred to a new tube. Toluidine blue solution (3 ml) and acetic acid solution (0.2 N) were then added to the tubes. The absorbance of the samples was measured at 630 nm using a microplate reader (Victor X3, PerkinElmer, Inc., USA).

The amount of inorganic phosphate in the medium absorbed by the cells was measured using a phosphate colorimetric analysis kit (MAK030; Sigma-Aldrich). Absorbance was measured in the same way as described above.

### Cultivation in Semi-Permeable Membrane Photobioreactor (SPM-PBR)

Similar to those in previous studies, *phoU* cells were cultured using cellulose-based semipermeable membranes (SpectraPor 3 Dialogis Membrane, Repligen, USA) [17]. Wild-type and Δ*phoU* cells were exposed to phosphate-deficient conditions for 3 days to induce phosphorus luxury uptake. They were then transferred to an SPM-PBR containing artificial wastewater to confirm phosphate removal. An acrylic rectangular reservoir (330 mm long × 230 mm wide × 145 mm high) capable of holding up to 5 L water was filled with 4 L artificial wastewater and shaken on a rocker to continuously mix the cells. To maintain a nutritional gradient, the artificial wastewater in the reservoir was replaced once per day. The reservoir was illuminated continuously at 30 μmol/m^2^/s with white LED lamps.

### Analysis of Total Phosphorus Content in Cells

The total intracellular phosphorus content of the Δ*phoU* strain was determined, using vanadate-molybdate reagent according to Standard Methods 4500-P [18], to assess the utility of this strain as a fertilizer in lettuce culture. Ammonium persulfate (0.4 g) was added to 1 ml of microalgal cell culture. The cells were then digested in an oven at 121°C for 30 min. The pH of cell lysates was then measured; if the pH was below 1, NaOH was added to adjust the pH to more than 7. After adjusting its total volume to 2 ml, the cell lysate was centrifuged at 2,350 ×*g* for 10 min, and then the supernatant was transferred to a new tube. The vanadate-molybdate reagent and cell lysates were mixed in equal proportions and allowed to react in the dark for 20 min at room temperature. Phosphorus concentrations were measured at 420 nm using a spectrophotometer (UV-1280, Shimadzu Corp., Japan).

### Culture and Measurement of Lettuce Plants

The lettuce (*Lactuca sativa* L.) seedlings used in this study were purchased from Gapjone Seedling Market (Republic of Korea). Temperature and light conditions were maintained in the growth culture chamber for constant adjustment, and water was supplemented by monitoring with a soil moisture meter. Lettuce seedlings were planted in 25 seedling pots and incubated at 500 μmol/m^2^/s for 10 days. The photoperiod was set for a 14 h/10 h light/dark cycle, and the temperature was 20°C.

The experimental design consisted of five culture groups defined by phosphorus concentration. The control group contained only water without the addition of other nutrients. Lettuce was treated with phosphorus fertilizer (0.157 mg P/m^2^); the Δ*phoU* cells contained the same phosphorus concentration as the phosphorus fertilizer. The compound fertilizer (0.0262 mg P/m^2^) and Δ*phoU* cells were added to the lettuce. After 10 days, the plants were harvested, and the number of leaves, height of lettuce, and dry weight were measured to confirm growth.

### Statistical Analysis

All experiments were performed in triplicate. Experimental data are expressed as the mean ± standard deviation of three measurements. All experimental results were analyzed in Microsoft Office Excel 365 (Microsoft, USA). The fresh cell weight and poly-P concentration were analyzed by Student’s *t*-test. In the lettuce culture experiment, five seedlings were planted for each condition, and the average of three measurements, excluding the highest and lowest values, were used in analyses. One-way analysis of variation (ANOVA) and *t*-test were employed to identify the significant differences between control and experimental groups.

## Results and Discussion

### Construction and Confirmation of the Δ*phoU* Strain

To prevent poly-P degradation, the *phoU* gene was knocked out of *Synechocystis* strain Syn6803. The *phoU* gene acts as a negative regulator of phosphate concentrations outside the cell. The plasmid was constructed by splitting *slr0741* into fragments 1 and 2 and inserting the spectinomycin resistance gene between them (Fig. 1A). A spectinomycin resistance cassette was inserted into *slr0741*, which encoded the *phoU* gene through double homologous recombination. The plasmid was transferred into the Syn6803 wild-type strain via natural formation, and the transformed cells were cultured in the BG-11 agar medium containing increasing concentrations of antibiotics for segregation. Thereafter, PCR and DNA sequencing were performed to confirm segregation (Fig. 1B). PCR was performed using primers for *phoU* fragment 1 - 1F and *phoU* fragment 2 – 2R. The band length of the wild type was 990 bp for the combination of fragments 1 and 2 and that of the transformant was 1,380 bp for *aadA*, larger than the band of the wild type. DNA sequencing was performed using the obtained bands to confirm the transformants.

**Fig. 2:**
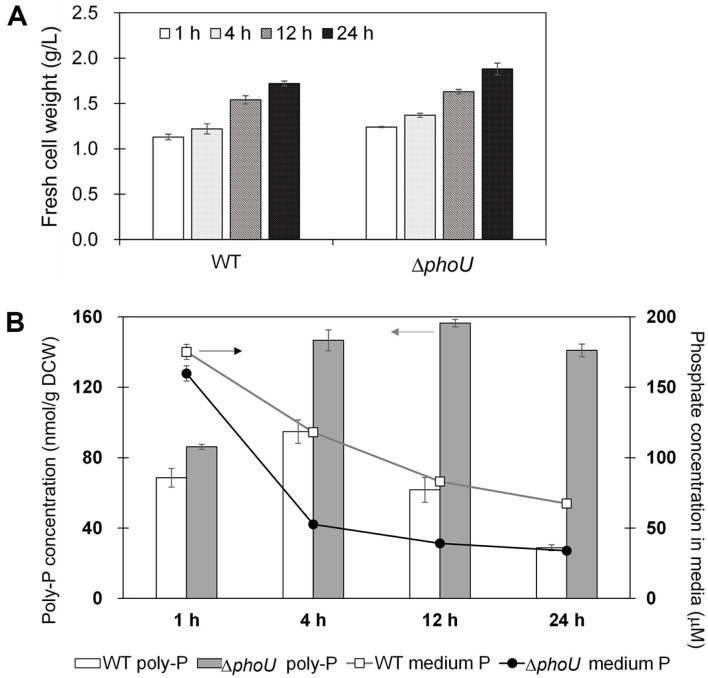
Growth and poly-P accumulation in BG-11 medium after the addition of excess phosphate (1.12 M). **A**. Fresh cell weight (g/l); **B**. poly-P (mM poly-P/g DCW) and remaining phosphate concentrations (mM) in media of wildtype and Δ*phoU* strains. Δ*phoU*: Syn6803 *phoU* knockout strain; poly-P, polyphosphate. Data are presented as the means of values obtained from experiments conducted in triplicate; standard deviation values are presented (vertical error bars). The fresh cell weight and poly-P concentration differed significantly: *p* < 0.05 by Student’s *t*-test.

The Δ*phoU* cells were cultivated in BG-11 medium to compare growth and the amount of poly-P with those in the wild type. Both the wild-type and Δ*phoU* strains showed similar growth, consistent with the results of previous studies [19]. When the FCW of the wild-type and Δ*phoU* strains reached 1 g/l, they were transferred to phosphate-free media to induce phosphorus uptake. After 72 h, K_2_HPO_4_ at a concentration of 1.12 mM, which has been shown to promote poly-P accumulation in previous studies, was added [20]. As shown in Fig. 2A, the growth of the wild-type and Δ*phoU* strains was similar, even after the addition of excess phosphate. Accumulated poly-P concentrations were highest in both the wild-type and Δ*phoU* strains at 4 h; poly-p concentration was more than 1.5 times higher in the Δ*phoU* strains than in the wild-type (Fig. 2B). While poly-P concentration in the Δ*phoU* strain showed a slight increase from 4 h up to 12 h, poly-P concentration in the wild type decreased by 69.6%compared to that in the wild type at 4 h. After 24 h of the addition of excess phosphate, poly-P concentration in the Δ*phoU* strain decreased by only 9.9% compared to that in the Δ*phoU* strain at 12 h. Phosphate concentration in the medium was rapidly reduced by the wild-type and Δ*phoU* strains to approximately 1 mM within 1 h of the addition of excess phosphate. After 12 h, both the wild-type and Δ*phoU* strains showed constant phosphate concentrations in the medium (Fig. 2B).

Poly-P degradation is regulated by the PhoU-negative regulatory protein [10]. PhoU inhibits the expression of proteins that absorb phosphate into cells and induces an increase in the expression of PPX proteins that break down intracellular poly-P when phosphate is present at high concentrations [9]. Production of the Δ*phoU* strain knocked out the negative regulator *phoU* gene, and this strain continuously absorbed high concentrations of externally present poly-P and accumulated higher concentrations of poly-P. The increase in poly-P concentration in this strain has been reported to result from improved expression of PstSCAB, a phosphate absorption system [10]. In addition, since poly-P concentration was maintained for 24 h after the addition of excess phosphate, it seems that PPX protein levels did not increase because of the absence of the PhoU protein. In contrast, when the *phoU* genes of *E. coli* and *Pseudomonas aeruginosa* are knocked out, inactivation of the phosphate absorption system PstSCAB results in a lower growth rate than that of the respective *E. coli* or *P. aeruginosa* wild type [21, 22]. Despite the knockout of *phoU* in Syn6803, however, the growth rate was similar to that of wild-type Syn6803. These findings suggest that the Δ*phoU* strain, which does not degrade accumulated poly-P, may be advantageous for replacing phosphorus fertilizer.

### Inhibition of Poly-P Degradation in Artificial Wastewater

**Fig. 3:**
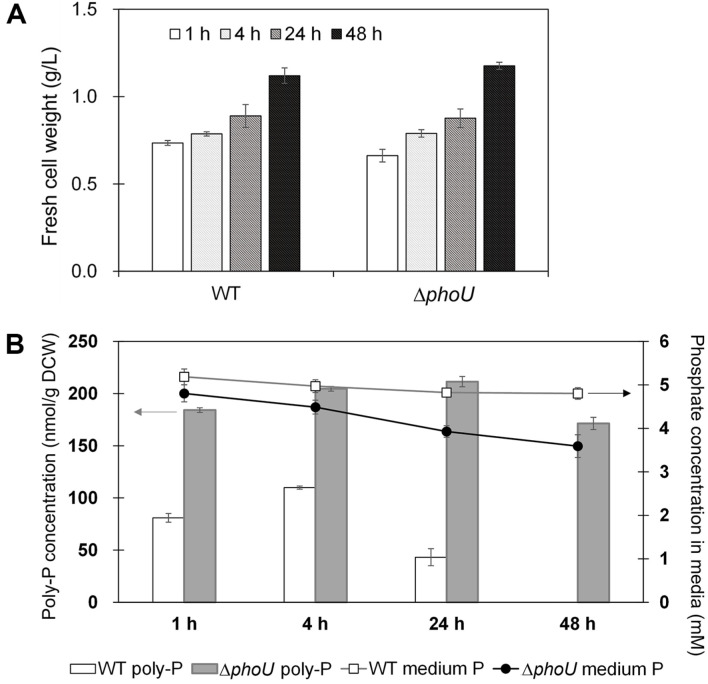
Growth and poly-P accumulation in artificial wastewater after the addition of excess phosphate (1.12M). **A**. Fresh cell weight (g/l); **B**. poly-P concentration (mM poly-P/g DCW) and remaining phosphate concentrations (mM) in media of the wild-type and Δ*phoU* strains. Δ*phoU*: Syn6803 *phoU* knockout strain; poly-P, polyphosphate. Data are presented as the means of values obtained from experiments conducted in triplicate; standard deviation values are presented (vertical error bars). Poly-P concentrations differed significantly: *p* < 0.05 by Student’s *t*-test.

The wild-type *phoU* and Δ*phoU* strains were cultured in artificial wastewater to confirm the applicability of the wastewater treatment process for phosphorus removal. Both the wild-type and Δ*phoU* strains showed growth patterns similar to those observed in BG-11 medium. In this experiment, when the FCW was approximately 1 g/l, the medium was replaced with a phosphate-deficient BG-11 medium and cultured for 72 h to induce phosphorus luxury uptake. After adding 6.9 mM phosphate, cell growth and the changes in poly-P accumulation were measured at 1, 4, 24, and 48 h. The FCW of the wild-type and Δ*phoU* strains increased after the addition of phosphate (Fig. 3A). The poly P concentration of the wild-type strain was highest at 4 h and that of the Δ*phoU* strain was highest at 24 h (Fig. 3B). The poly P concentration in the Δ*phoU* strain accumulated to 2.5 times that in the wild type, even at 4 h, when poly-P concentration in the wild type was the highest. Phosphate concentration in the medium was such that both the wild-type and Δ*phoU* strains consumed more than 99% of the 6.9 mM excess phosphate added after phosphate depletion within 1 h. The concentration of phosphate in the wild-type medium decreased after 4 h of the addition of phosphate and then remained constant until 24 h (Fig. 3B). In contrast, phosphate concentration in the Δ*phoU* strain medium decreased sharply up to 4 h and then decreased gradually to 16.2% over 24 h.

In conventional wastewater treatment processes, a group of heterotrophs, phosphate-accumulating organisms (PAOs), is used to remove phosphorus. Removal of phosphorus by PAO results in the accumulation of a large amount of poly-P in cells under aerobic conditions, but this process requires the supply of costly organic carbon sources [23, 24]. In contrast, microalgae acquire inorganic carbon sources through photosynthesis, and inorganic/organic nitrogen in wastewater can be removed along with phosphorus. Studies have reported the removal of nitrogen and phosphorus from microalgae such as *Chlamydomonas*, *Chlorella*, and *Scenedesmus* in wastewater [25-27]. In the case of *Anabaena* PCC7120 with the *phoU* gene removed, 96.9% of phosphate was removed from aquaculture water containing 7.9 mg P/L; in the case of *Synechocystis*, 8 mg P/L within 5 h was removed, similar to the results of this experiment [11, 28].

**Fig. 4:**
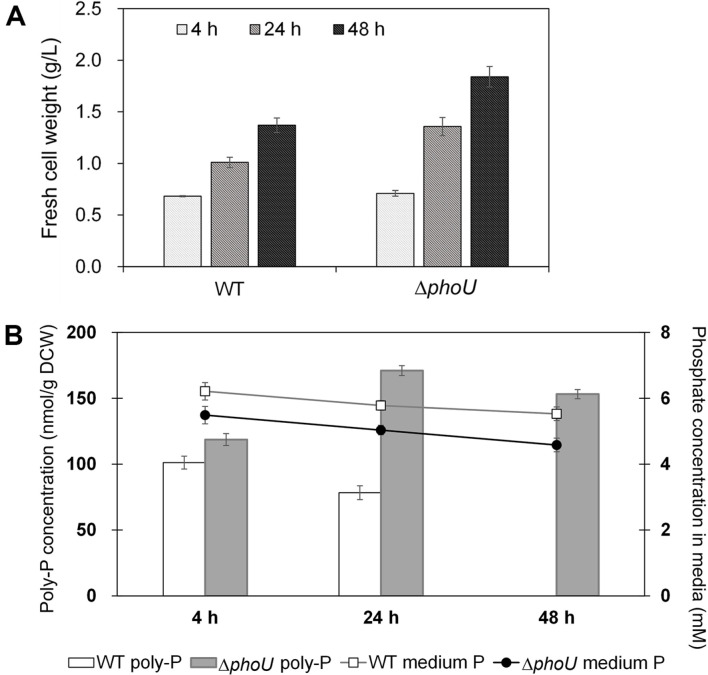
Growth and poly-P accumulation in a semi-permeable photobioreactor after the addition of excess phosphate (1.12 M). **A**. Fresh cell weight (g/l); **B**. poly-P concentration (mM poly-P/g DCW) and remaining phosphate concentrations (mM) in media of the wild type and Δ*phoU*. Δ*phoU*: Syn6803 *phoU* knockout strain; poly-P, polyphosphate. Data are presented as the means of values obtained from experiments conducted in triplicate; standard deviation values are presented (vertical error bars). Poly-P concentrations differed significantly: *p* < 0.05 by Student’s *t*-test.

Poly-P accumulation was confirmed by employing a semi-permeable photoreactor used to evaluate the eutrophication of rivers or to remove or recover nitrogen and phosphorus from agricultural water. As in a previous study, wild-type and Δ*phoU* cells were cultured under phosphate-deficient conditions at the flask scale, transferred to a semi-permeable membrane, and cultured in a water reservoir containing artificial wastewater. The biomass of the Δ*phoU* strain was 34.3% higher than that of the wild type at 48 h (Fig. 4A). Poly-P accumulation in the wild type was highest at 4 h, and poly-P was completely degraded at 48 h. In contrast, the poly-P concentration in Δ*phoU* strains was highest at 24 h and decreased to 10.5% at 48 h. The difference in growth and poly-P accumulation between flasks and the SM-PBR seems to be due to the rate at which nutrient ions diffuse through the semipermeable membrane [17]. The SM-PBR did not significantly affect the growth rate of the Δ*phoU* strain or poly-P accumulation, which suggests that the phosphate dissolved in rivers, agricultural water, and livestock wastewater can be captured using microalgae. As more than 80% of the phosphorus used in agriculture is discharged into water, the phosphorus discharged by microalgae from the SM-PBR can be recovered for use in agriculture again [29].

### Effect of Mutants as a Phosphorus Fertilizer on Lettuce

**Fig. 5:**
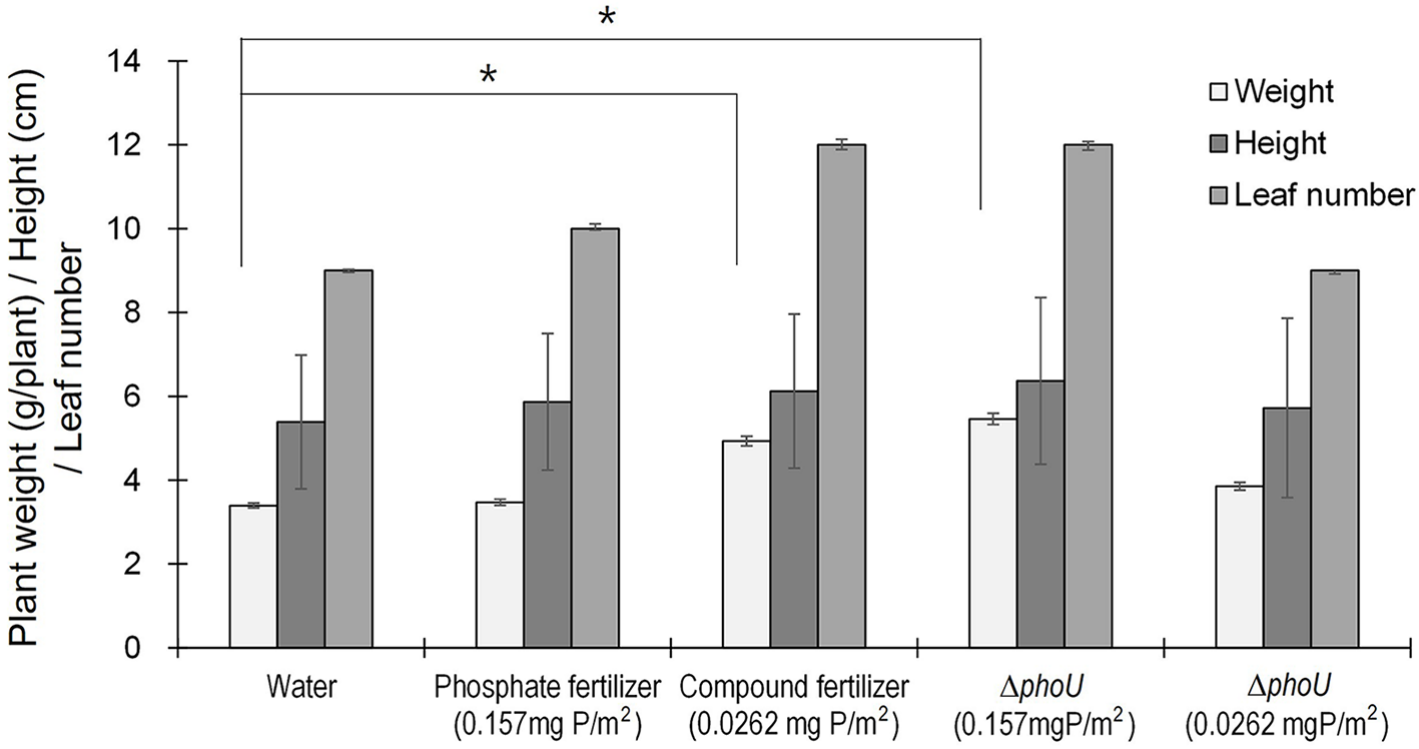
Comparison of height, weight, and number of leaves of lettuce before and after treatment with chemical fertilizers and Δ*phoU* biomass as a biofertilizer. Δ*phoU*: Syn6803 *phoU* knockout strain; poly-P, polyphosphate. Weight and leaf number differed significantly, as evidenced by one-way ANOVA (*p* < 0.0001). Height did not show a statistically significant difference (*p* > 0.05). Comparison between Δ*phoU* of fertilizer-treated groups with the water group by Student’s *t*-test. Data are presented as the mean±standard deviation. Asterisks (*) on the bars indicate a statistically significant difference (*p* < 0.05).

An experiment was conducted to confirm whether the phosphate in Δ*phoU* strain could replace phosphate fertilizers and promote the growth of edible crops. Cyanobacteria, including wild-type *Synechocystis*, are beneficial to crop productivity [30]; therefore, we checked if Δ*phoU* strain, a knockout, could allow nutrients to move out of the cell and make them available as fertilizer. Lettuce was used as an edible crop product, and Δ*phoU* strains were cultivated with commercial fertilizers to determine whether they could be used as biofertilizers. Fig. 5 shows the relative differences in dry weight, leaf size, and the number of leaves between the control and experimental groups. All experimental groups had higher dry weights, higher leaf heights, and higher numbers of leaves than those in the control group. The weight in the phosphate fertilizer (0.157 mg P/m^2^)- and Δ*phoU* strain (0.0262 mg P/m^2^)-treated groups were not significantly different from that of the control, showing an increase of 2% and 12%, respectively. In comparison, the weight of the Δ*phoU* strain (0.157 mg P/m^2^) was the highest, showing a 38% increase from that of the control. The compound fertilizer contained 0.0262 mg P/m^2^, but the weight of the lettuce increased by 31% compared to that of the control. Therefore, the growth limiting factor for Δ*phoU* (0.0262 mg P/m^2^)- and the phosphate fertilizer (0.157 mg P/m^2^)-treated groups may not be phosphorus. This seems to show growth similar to that achieved using compound fertilizers, as the nitrogen required for plants is also present in the Δ*phoU* strain (0.157 mg P/m^2^) as a biomass component of microalgae. Our study shows that the addition of Δ*phoU* (0.157 mg P/m^2^) biomass significantly improves plant growth. Dry weight and leaf number significantly increased after the addition of Δ*phoU* (0.157 mg P/m^2^) biomass. Differences between the control and Δ*phoU* strain (0.157 mg P/m^2^)-treated groups indicated that nutrients, including phosphorus in microalgae, were utilized by lettuce plants for their growth. These results are similar to those of a previous report on microorganisms used in plants. Rice cultivation by inoculating the plants with *Chlorella* and *Spirulina* increased rice yields by up to 20.9% [31]. In addition, maize plant growth was improved by up to 51.1% when microalgae mixed with cow manure were used to treat maize plants [32]. Treatment of tomato plants with dried cells of *Acutodesmus dimorphus*, a green alga, showed positive effects on seed germination, plant growth, and fruit production [33].

Microalgae have the advantage of accumulating poly-P from excess phosphorus and slowly releasing it back into the soil in the form of phosphate, which suggests the possibility of the use of microalgae as a fertilizer [6]. This is because the loss of phosphorus can be prevented by delaying or controlling phosphorus release into the soil by reducing leaching, volatilization, and adsorption by soil particles [34]. Microalgae also interact with other soil microorganisms to enhance soil fertility and increase plant growth and crop yield [35]. In particular, because the *phoU* gene can be induced or repressed by quorum-sensing signals from bacteria, the development of fertilizers along with other microorganisms to improve their performance is a potential strategy [36]. Therefore, if phosphorus recovered from agricultural wastewater by microalgae is used to improve plant growth and fertilize soils, the phosphorus circulation process can be restored.

## Conclusion

In this study, a mutation in the *phoU* gene allowed poly-P accumulation and maintenance without degradation in *Synechocystis* cells. In BG-11 medium, a large amount of poly-P accumulation was continuously maintained in the Δ*phoU* strain, but not in the wild-type strain. In addition, artificial wastewater experiments showed that the poly-P decomposition rate was low, with the concentration of poly-P maintained for up to 24 h and decreasing by 16.2% after 48 h. This suggests that poly-P concentration remains constant over time through knockout of the *phoU* gene compared to that in the wild-type strain, wherein poly-P concentration decreases rapidly within 48 h. Lettuce experiments confirmed that cells with accumulated phosphorus can replace commercial fertilizers. The results of this study can be used as a basis for additional genetic engineering to increase intracellular poly-P levels. Furthermore, this study, demonstrating the applicability of a microalgae-based biofertilizer, would contribute to the establishment of a sustainable agricultural system.

## Figures and Tables

**Table 1:** Sequences of nucleic acid primers used for plasmid construction.

Primer	Nucleotide sequence (5'→3')
*phoU* fragment 1 - 1F	AAAACGACGGCCAGTGAATTCAATGAACACACCAATTCTCCATGGA
*phoU* fragment 1 - 1R	GTTCGCCCAGCCCCCAAATCCTGGGCAT
*aadA* - 2F	GATTTGGGGGCTGGGCGAACAAACGATGC
*aadA* - 2R	ATGGCAATTTCGTCGGCTTGAACGAATTG
*phoU* fragment 2- 3F	CAAGCCGACGAAATTGCCATGAAGTTGACCCG
*phoU* fragment 2 - 3R	TACGCCAAGCTTGCATGCCTGCAGGAATACAATTGGGCATAAAAAAAGC

## References

[1] Lu C, Tian H (2017). Global nitrogen and phosphorus fertilizer use for agriculture production in the past half century: shifted hot spots and nutrient imbalance. Earth Syst. Sci. Data.

[2] Smil V (2002). Nitrogen and food production: proteins for human diets. Ambio.

[3] Beardsley TM (2011). Peak phosphorus. Bioscience.

[4] Cordell D, White S (2011). Peak phosphorus: clarifying the key issues of a vigorous debate about long-term phosphorus security. Sustainability.

[5] Solovchenko A, Verschoor AM, Jablonowski ND, Nedbal L (2016). Phosphorus from wastewater to crops: an alternative path involving microalgae. Biotechnol. Adv..

[6] Solovchenko AE, Ismagulova TT, Lukyanov AA, Vasilieva SG, Konyukhov IV, Pogosyan SI (2019). Luxury phosphorus uptake in microalgae. J. Appl. Phycol..

[7] Powell N, Shilton AN, Pratt S, Chisti Y (2008). Factors influencing luxury uptake of phosphorus by microalgae in waste stabilization ponds. Environ. Sci. Technol..

[8] Brown N, Shilton A (2014). Luxury uptake of phosphorus by microalgae in waste stabilisation ponds: current understanding and future direction. Rev. Environ. Sci. Bio. Technol..

[9] Munévar NFV, de Almeida LG, Spira B (2017). Differential regulation of polyphosphate genes in *Pseudomonas aeruginosa*. Mol. Genet. Genomics.

[10] Morohoshi T, Maruo T, Shirai Y, Kato J, Ikeda T, Takiguchi N (2002). Accumulation of inorganic polyphosphate in *phoU* mutants of *Escherichia coli* and *Synechocystis* sp. strain PCC6803. Appl. Environ. Microbiol..

[11] Burut-Archanai S, Eaton-Rye JJ, Incharoensakdi A, Powtongsook S (2013). Phosphorus removal in a closed recirculating aquaculture system using the cyanobacterium *Synechocystis* sp. PCC 6803 strain lacking the *SphU* regulator of the Pho regulon. Biochem. Eng. J..

[12] Krasaesueb N, Promariya A, Raksajit W, Khetkorn W (2021). Inactivation of phosphate regulator (SphU) in cyanobacterium *Synechocystis* sp. 6803 directly induced acetyl phosphate pathway leading to enhanced PHB level under nitrogen-sufficient condition. J. Appl. Phycol..

[13] Mukherjee C, Chowdhury R, Ray K (2015). Phosphorus recycling from an unexplored source by polyphosphate accumulating microalgae and cyanobacteria - A step to phosphorus security in agriculture. Front. Microbiol..

[14] Rippka R, Deruelles J, Waterbury JB, Herdman M, Stanier RY (1979). Generic assignments, strain histories and properties of pure cultures of cyanobacteria. Microbiology.

[15] Kim ZH, Kim SH, Lee HS, Lee CG (2006). Enhanced production of astaxanthin by flashing light using *Haematococcus pluvialis*. Enzyme Microb. Technol..

[16] Eaton-Rye JJ, Carpentier R (2004). The construction of gene knockouts in the cyanobacterium *Synechocystis* sp. PCC 6803. Photosynthesis research protocols.

[17] Kim ZH, Park H, Ryu YJ, Shin DW, Hong SJ, Tran HL (2015). Algal biomass and biodiesel production by utilizing the nutrients dissolved in seawater using semi-permeable membrane photobioreactors. J. Appl. Phycol..

[18] Rice EW, Bridgewater L, Association APH (2012). Standard methods for the examination of water and wastewater.

[19] Juntarajumnong W, Hirani TA, Simpson JM, Incharoensakdi A, Eaton-Rye JJ (2007). Phosphate sensing in *Synechocystis* sp. PCC 6803: SphU and the SphS-SphR two-component regulatory system. Arch. Microbiol..

[20] Voronkov A, Sinetova M (2019). Polyphosphate accumulation dynamics in a population of *Synechocystis* sp. PCC 6803 cells under phosphate overplus. Protoplasma.

[21] de Almeida LG, Ortiz JH, Schneider RP, Spira B (2015). *phoU* inactivation in *Pseudomonas aeruginosa* enhances accumulation of ppGpp and polyphosphate. Appl. Environ. Microbiol..

[22] Steed P, Wanner B (1993). Use of the rep technique for allele replacement to construct mutants with deletions of the *pstSCAB-phoU* operon: evidence of a new role for the PhoU protein in the phosphate regulon. J. Bacteriol..

[23] Guerrero J, Guisasola A, Baeza JA (2015). Controlled crude glycerol dosage to prevent EBPR failures in C/N/P removal WWTPs. Chem. Eng. J..

[24] Melia PM, Cundy AB, Sohi SP, Hooda PS, Busquets R (2017). Trends in the recovery of phosphorus in bioavailable forms from wastewater. Chemosphere.

[25] Kong Q-x, Li L, Martinez B, Chen P, Ruan R (2010). Culture of microalgae *Chlamydomonas reinhardtii* in wastewater for biomass feedstock production. Appl. Biochem. Biotechnol..

[26] Voltolina D, Cordero B, Nieves M, Soto LP (1999). Growth of *Scenedesmus* sp. in artificial wastewater. Bioresour. Technol..

[27] Wang C, Yu X, Lv H, Yang J (2013). Nitrogen and phosphorus removal from municipal wastewater by the green alga *Chlorella* sp. J.Environ.Biol..

[28] Burut-Archanai S, Powtongsook S (2017). Identification of negative regulator for phosphate-sensing system in *Anabaena* sp. PCC 7120: a target gene for developing phosphorus removal. Biochem. Eng. J..

[29] Hussain F, Shah SZ, Ahmad H, Abubshait SA, Abubshait HA, Laref A (2021). Microalgae an ecofriendly and sustainable wastewater treatment option: biomass application in biofuel and bio-fertilizer production. A review. Renew. Sust. Energ. Rev..

[30] Singh JS, Kumar A, Rai AN, Singh DP (2016). Cyanobacteria: a precious bio-resource in agriculture, ecosystem, and environmental sustainability. Front. Microbiol..

[31] Dineshkumar R, Kumaravel R, Gopalsamy J, Sikder MNA, Sampathkumar P (2018). Microalgae as bio-fertilizers for rice growth and seed yield productivity. Waste Biomass Valori..

[32] Dineshkumar R, Subramanian J, Gopalsamy J, Jayasingam P, Arumugam A, Kannadasan S (2019). The impact of using microalgae as biofertilizer in maize (*Zea mays* L.). Waste Biomass Valori..

[33] Garcia-Gonzalez J, Sommerfeld M (2016). Biofertilizer and biostimulant properties of the microalga *Acutodesmus dimorphus*. J. Appl. Phycol..

[34] Chen J, Lü S, Zhang Z, Zhao X, Li X, Ning P (2018). Environmentally friendly fertilizers: a review of materials used and their effects on the environment. Sci. Total Environ..

[35] Guo S, Wang P, Wang X, Zou M, Liu C, Hao J, Alam MA, Xu JL, Wang Z (2020). Microalgae as biofertilizer in modern agriculture. Microalgae biotechnology for food, health and high value products.

[36] Ren D, Bedzyk LA, Ye RW, Thomas SM, Wood TK (2004). Stationary-phase quorum-sensing signals affect autoinducer-2 and gene expression in *Escherichia coli*. Appl. Environ. Microbiol..

